# Coping Strategies Before Competition: The Role of Stress, Cognitive Appraisal, and Emotions

**DOI:** 10.3390/sports13100366

**Published:** 2025-10-16

**Authors:** José Miguel Nogueira, Clara Simães, Catarina Morais, Paul Mansell, A. Rui Gomes

**Affiliations:** 1 Adaptation, Performance and Human Development Research Group, School of Psychology, University of Minho, 4710-057 Braga, Portugal; jmgama@sapo.pt; 2 Health Sciences Research Unit: Nursing (UICISA: E), Nursing School of Coimbra (ESEnfC), 3046-851 Coimbra, Portugal; csimaes@ese.uminho.pt; 3 School of Nursing, University of Minho, 4710-057 Braga, Portugal; 4 Research Centre for Human Development, Faculty of Education and Psychology, Universidade Católica Portuguesa, 1649-023 Porto, Portugal; ctmorais@ucp.pt; 5 School of Health, Education, Policing and Sciences, Department of Sport and Exercise, University of Staffordshire, Stoke-on-Trent ST4-2DE, UK; paul.mansell@staffs.ac.uk; 6 Psychology Research Centre, School of Psychology, Campus de Gualtar, University of Minho, 4710-057 Braga, Portugal

**Keywords:** sports, competition, athletes, psychological factors, challenge, gender

## Abstract

Sports, and especially competitions, can be a stressful experience for athletes, who often struggle to find and apply strategies to cope with stress. Thus, this study analyzes how different coping strategies anticipated to be employed in an important competition are explained by psychological (i.e., cognitive appraisal and emotions) and person and sports-related factors (i.e., gender, type of sport). Specifically, athletes were asked to complete a protocol 24–48 h prior to an important competition to assess their adaptation to stress related to high performance. The study included 383 athletes (60% male, *M*_age_ = 22.9 ± 5.3 years), from individual (swimming and running, *n* = 157; 41%) and team sports (handball, volleyball, *n* = 226; 59%) competing in major national leagues. Hierarchical linear regression analyses (enter method) were performed to examine the extent to which coping strategies and coping efficacy were explained by psychological, personal and sport-related variables. Results indicated (a) higher control perception and excitement were related with higher intention to use active coping; (b) being a female athlete, practicing individual sports, and excitement (higher intensity and facilitative value) were associated with a higher intention to use emotional support; (c) being a female athlete, lower coping perception, higher anger intensity, and higher facilitative value of happiness were associated with a higher anticipated use of humor; and (d) being a male athlete, higher anxiety, anger, and happiness intensity, and lower facilitative value of dejection and excitement were associated with higher anticipated use of denial. In sum, the explanation of each coping strategy is distinct and should be analyzed separately.

## 1. Introduction

Participating in sport can be a very stressful experience, with athletes reporting a wide variety of stressors. Common stressors include pressure from coaches, the expectations of others (e.g., fans, family, friends, teammates), organizational aspects, or extra-sporting situations; and an important emphasis has been given to competition-related stressors, especially not achieving the desired performance, e.g., [1,2,3,4,5,6]. Based on the numerous sources of stress athletes face throughout their careers, and especially during competition, understanding how athletes manage these stressors is central to studying adaptation and performance under pressure. Stress management relies on athletes’ ability to employ strategies to deal with the situation, that is, their ability to cope with the stressful situations. Coping is, therefore, a key concept in this context and it is closely related to how individuals perceive (cognitive appraisal) and experience (emotions) the stressful situation [7]. Coping may be defined as “constantly changing cognitive and behavioural efforts to manage specific external and/or internal demands that are appraised as taxing or exceeding the resource of the person” [8], p. 141. In sport, coping strategies aim to help athletes manage the emotional and cognitive responses triggered by stress, facilitating successful performance [9].

Coping is recognized as a crucial factor for athletes’ performance and satisfaction, representing the ability to manage stressors through voluntary cognitive and behavioral actions [8]. Various classifications exist, ranging from general frameworks to sport-specific models (see Nicholls et al. [5] for a review). In this study, we focus on three main dimensions: (1) Problem-focused coping—efforts to change or actively manage the stressful situation (e.g., planning, increased effort) [8,10]; (2) Emotion-focused coping—strategies to regulate emotional responses (e.g., reappraisal, relaxation [7]), further divided into active and passive approaches, with passive strategies generally linked to negative outcomes and active strategies associated with better performance [8,10]; and (3) Seeking social support—involving others’ assistance, recognized as central in most conceptual models [11]. This distinction between active and passive approaches is fundamental, as literature suggests that the use of more passive strategies translates into more negative effects for the person, in terms of health and wellbeing, as well as worse adjustment to stress [12], while active emotional regulation is associated with increased sport performance [13]. Literature has also examined coping efficacy, highlighting its role in athletes’ adaptation to stress: effective coping has been associated with improved performance, while ineffective coping can impair performance and increase dropout risk [2,14]. Building on this, we now consider the psychological factors that influence the adoption and effectiveness of coping strategies.

### 1.1. Psychological Factors and Coping

Transactional models of stress recognize that stress results from the interaction between the individual and the environment [8,9,11]. An event is considered stressful if the individual appraises it as important and potentially harmful. Lazarus’ cognitive-motivational-relational (CMR) model provides a framework for understanding these processes, emphasizing that coping, appraisal, and emotions should be considered together for a complete understanding of stress adaptation. Specifically in the sports context, there have been attempts to analyze the various factors of the adaptive process together, which have resulted in theoretical perspectives that are conceptually close to Lazarus’ transactional approach, e.g., [15,16,17]. However, the need remains for additional studies that contribute to a more detailed understanding of the stress process may occur [1,5,15,18,19].

Cognitive appraisal guides how athletes interpret a situation and their coping options. Primary appraisal involves evaluating whether a situation poses threat, loss, challenge or benefit, whereas secondary appraisal assesses coping resources and control [8,20,21]. Speaking only of the types of evaluation that focus on the future, threat occurs when the situation is evaluated as being potentially negative, generating damage and fear of failure. In challenge appraisals, a potential gain is attributed to the situation, even if the difficulty of achieving it is recognized, meaning that the athlete is more focused on seeking personal progress and achieving success [20]. In the secondary cognitive appraisal, beyond the perception of coping which is related to the perceived ability to combat adversity, it also includes the perception of control, linked to the understanding that it depends (or not) on the athlete to manage and reduce stress levels [9,20]. An important aspect is that greater personal control over the situation explains the more regular use of problem-solving strategies, which research has associated with higher levels of performance [22]. Emotions also play a critical role. Beyond intensity, the perceived effect of emotions on performance—termed emotion direction—influences coping choices. This issue is important if we want to understand the profile of each emotional pattern in sporting contexts. Athletes who perceive emotions as facilitative are more likely to engage in adaptive coping strategies [23,24,25,26,27,28,29]. Having outlined psychological mechanisms, we now turn to personal and sport-related factors that shape coping.

### 1.2. Personal and Sport-Related Variables

Furthermore, research has shown that certain personal and sporting factors can play an important role in how stressful situations are managed. For example, there is evidence that older athletes may use problem-focused coping more effectively due to accumulated experience and more functional appraisal strategies [30] and male athletes opt more for problem-focused strategies, confirming that women resort more regularly to emotion regulation strategies [22,31]. The type of sport (e.g., individual, team) should also be considered a relevant factor in terms of the different dimensions of coping and the effectiveness attributed to stress management efforts. Sport type (individual vs. team) also impacts coping strategies: individual athletes often rely on problem-solving approaches, whereas team athletes show higher engagement in social support and collective strategies, e.g., [32,33,34]. In team contexts, shared coping strategies—such as collective emotional regulation and relationship-focused strategies—emerge as key for managing shared stressors. These empirical findings justify the inclusion of personal and sport-related variables as moderators in the study of coping, while highlighting the need for further investigation, especially regarding team vs. individual sport distinctions. With these theoretical and empirical foundations, we can now specify the study’s objectives.

### 1.3. Study Rationale and Goals

The main objective of this study was to analyze the adaptation of athletes to a sport-related stressor (not achieving the desired performance in the next competition), assessing which psychological variables (e.g., overall stress, cognitive appraisal, and intensity and direction of emotions) explain the coping strategies that athletes anticipate using to manage stress. This analysis was performed by controlling athletes’ personal (e.g., sex and age) and sporting (e.g., type of sport) characteristics. Specifically, we aimed to examine whether the coping dimensions assessed in this study are stronger indicators of successful adaptation to a stressful situation—defined here as failing to achieve the desired performance in the upcoming competition. Thus, the analysis of adaptation to stress is carried out under the logic of critical incident see [35,36], as athletes report their intended use coping strategies when confronted with a potential stressful situation that can arise in the upcoming competition and that can jeopardize the achievement of their goals.

Therefore, we sought to assess whether coping strategies (active coping, emotional support, humor, and denial) were explained by overall stress, cognitive appraisal, and emotions that athletes feel prior to a competition. In this way, the main aim of this study is to contribute to understanding the process of human adaptation to sport in an integrated way, on the factors associated with coping strategies.

In sum, we hypothesize that athletes’ coping strategies are associated with psychological factors and sport-related and personal factors are covariates related to coping process. Specifically, we expect problem-focused coping and higher coping efficacy to relate more strongly to psychological variables closely linked to successful sport adaptation, such as positive emotions, facilitative emotion direction, and functional cognitive appraisal dimensions (challenge, coping perception, and control) [3,7,16,18,19]. To the best of our knowledge, there is not enough knowledge about the factors explaining coping strategies used by athletes to deal with specific stressors prior to competition.

## 2. Materials and Methods

### 2.1. Participants

The sample for this study consisted of 383 athletes (154 females, 40%; 229 males, 60%) between 14 and 37 years old (*M* = 22.85; *SD* = 5.35). Athletes were recruited from team sports (59%; 125 handball and 101 volleyball players) and individual sports (41%; 105 swimmers and 52 runners). Their practice experience ranged between 3 and 27 years (*M* = 11.65; *SD* = 4.93). They were all competing at major leagues of their respective competitions (underage athletes were also part of top-level teams). At the time of data collection, 61% (*n* = 234) were competing for the national championship, 34% to avoid relegation, and 5% competing at European and World-level competitions. Most athletes had a record of at least one national title (72%), had represented their national team (55%), and an average of 17 international appearances for their country (*SD* = 33.7).

### 2.2. Procedure and Study Design

The present research followed cross-sectional and correlational design and was conducted during one competitive season and involved the collection of primary data through validated self-report questionnaires. The design aimed to examine the relationships between athletes’ coping strategies and psychological, personal, and sport-related factors.

Ethical approval was granted by the Ethics Committee for Research in Social and Human Sciences of the Ethics Council of the University of Minho (References 026/2014). Clubs that had high-level teams and athletes, competing at the major leagues and competitions, were contacted to participate in the study. A total of 37 clubs agreed to allow the research team to meet with team managers (for team sports) and coaches (for individual sports) to ask for permission to approach athletes. Once authorized, one of the members of the research team arranged a meeting with athletes at each club for recruitment purposes. Participants were then informed about the aims of the study and were assured of confidentiality and anonymity, with the option to withdraw at any time without penalty. No incentives or rewards were offered for participation. Those who agreed to participate (and their legal guardians, in the case of underage athletes) signed informed consent—the participation rate was 92% (383 participants out of 415).

Athletes completed a set of questionnaires at the facilities of their club and in the presence of the first author. The average time to complete the protocol was approximately 20 min. The questionnaire was completed 24 to 48 h before the upcoming competition, using a critical incident approach. The competitions selected for data collection were the final stages of the season—with athletes competing for the final classifications of their championships—or knockout stages of national cups. By selecting these specific competitive moments, the research team aimed to ensure that all athletes were exposed to highest levels of performance-related stress.

### 2.3. Measures

Emotions. Using the Sport Emotional Questionnaire (SEQ; [29]; translated to Portuguese by Gomes et al. [37]), athletes were presented with five different sets of emotions related to how they feel regarding the next competition. For each emotion, they rated the *intensity* (0 = *Not at all*; 4 = *Extremely*) and *direction* in terms of helpful/unhelpful role of that emotion (−3 = *Very negative*; 0 = *Indifferent*; +3 = *Very positive*) to the performance in the upcoming competition. Their responses were averaged to calculate, for each emotion, one intensity and one direction score [anxiety (5 items; α_intensity_ = 0.86; α_direction_ = 0.80), dejection (5 items; α_intensity_ = 0.88; α_direction_ = 0.91), anger (4 items; α_intensity_ = 0.69; α_direction_ = 0.72), excitement (4 items; α_intensity_ = 0.86; α_direction_ = 0.88), and happiness (4 items; α_intensity_ = 0.93; α_direction_ = 0.87)]. Confirmatory-factor analyses showed adequate fit for both *intensity* [*χ*^2^ (198) = 459.74, *p* < 0.001; RMSEA = 0.059, 90% C.I. [0.052; 0.066]; CFI = 0.95; NFI = 0.91; TLI = 0.94) and *direction* [*χ*^2^ (199) = 460.55, *p* < 0.001; RMSEA = 0.059, 90% C.I. [0.052; 0.066]; CFI = 0.94; NFI = 0.91; TLI = 0.94].

Cognitive Appraisal. Athletes were asked to complete the Primary and Secondary Cognitive Appraisal Scale (PSCAS; [38]), considering the upcoming competitions. Using a 7-point Likert scale, athletes indicated to which extent they perceived the upcoming competition as stimulating/positive (*challenge perception*, three items, α = 0.61) or threatening/negative (*threat perception,* three items, α = 0.64), to their athletic abilities. Additionally, they evaluated to which extent they perceived to have the abilities/resources (*coping perception,* three items, α = 0.84) and control (*control perception,* three items, α = 0.86) over the competition’s demands. Their responses were averaged to calculate a score for each dimension, so higher scores meant higher evaluations. A confirmatory-factor analysis indicated the expected factor structure was a good fit to the data [*χ*^2^ (48) = 165.85, *p* < 0.001; RMSEA = 0.080, 90% C.I. [0.067; 0.094]; CFI = 0.93; NFI = 0.90; TLI = 0.90].

Coping. The Reduced Coping Inventory (Coping-R; [39]), is divided into two parts. In the first one, athletes indicate *the overall stress* (1 = *low stress*, 5 = *high stress*) caused by a particular situation. In the case of this study, such stressful situation was defined as “not achieving the desired performance in the upcoming competition”. In the second part, they rated their intention to use (1 = *I will never use it*; 5 = *I will use it often*) four different coping strategies to deal with the described stressful situation (anticipatory-specific version): active coping (α = 0.81), emotional support (α = 0.90), humor (α = 0.83), and denial (α = 0.65). Confirmatory-factor analysis showed a good fit of this structure to the data, [*χ*^2^ (98) = 197.06, *p* < 0.001; RMSEA = 0.051, 90% C.I. [0.041; 0.062]; CFI = 0.96; NFI = 0.93; TLI = 0.95].

### 2.4. Data Analysis Strategy

Statistical analyses were conducted using IBM SPSS Statistics 29. The first step consisted of checking normality and multicollinearity assumptions. Then, descriptive analyses and Pearson correlations were conducted to summarize data and assess relationships between variables. To ensure that athletes perceived the situation of “not achieving the desired performance in the upcoming competition” as stressful, a One-sample t-test was conducted. To test the study hypothesis, hierarchical linear analyses using the Enter Method were performed for each dependent variable (coping strategy). The same procedure was followed in all of them: in the first block, personal and sport-related variables were included (i.e., gender, age, type of sport). In the second block, we controlled for overall perceived stress (Coping-R). In the third, fourth and fifth block, the psychological variables of cognitive appraisal, emotions’ intensity and emotions’ direction were included. The final sixth block included the interaction between Emotional Intensity and Direction interaction.

## 3. Results

### 3.1. Statistical Assumptions

Normality assumptions were assessed using the cut-off points defined by Kline [40] of skewness (|3|) and kurtosis (|10|). Non-multicollinearity assumptions were met if correlations < 0.80 and Variance Inflation Factor < 5 (VIF; cf. [41]). Table 1 shows these assumptions were met for all variables of the study and, therefore, parametric tests could be conducted to test the aims of this study. Full descriptive statistics comparing the different variables of the study based on Sex and Type of sport are presented in Table S1. Moreover, to ensure that athletes perceived the idea of not performing as expected in the upcoming competition as stressful, a one-sample *t*-test was conducted confronted athletes’ overall stress rate with the mid-point of the scale (3 = *somewhat stressful*). Results indicated that, as expected, athletes perceived the situation as stressful, as their average rating was significantly above the mid-point, *t*(382) = 6.29, *p* < 0.001, *d* = 0.32.

### 3.2. Regression Analysis of Coping Strategies

Hierarchical regression analyses were conducted to test which variables were related to the use of different coping strategies. Therefore, for each coping strategy, the following variables were included in the model: sex, age, and type of sport in block 1; overall stress in block 2; cognitive appraisal in block 3; intensity of emotions in block 4; direction of emotions in block 5; and the interaction between intensity and direction of emotions in block 6. Table 2 graphically summarizes the main results and full statistical coefficients for block 6 are displayed in Table 3 (and step-by-step results can be accessed in Table S2).

#### 3.2.1. Active Coping

Higher perceptions of control and higher intensity of excitement were associated with a stronger intention to use active coping to deal with stress in the upcoming competition. Neither primary cognitive appraisal nor the direction of emotions emerged as significantly associated variables in the final block.

#### 3.2.2. Emotional Support

Demographic variables assumed a key role in explaining the use of emotional support: female athletes and individual sports’ athletes anticipated a more frequent use of emotional support compared to male and collective sport athletes. Regarding emotions, only the association to excitement emerged as significant: athletes showed higher use of emotional support when they felt stronger intensity of excitement and when such excitement was perceived as mostly unhelpful for performance (this result is also qualified by a marginally significant intensity and direction interaction—cf. Table 2).

#### 3.2.3. Humor

Opposite to emotional support, humor was mostly used by male athletes. Regarding cognitive appraisal, lower perceptions of coping was related to higher use of humor. More intense feelings of anger, as well as perceptions that anger (marginally significant) and happiness might be more detrimental to performance, were related to higher use of humor as a coping strategy.

#### 3.2.4. Denial

The intensity of emotions prior to the competition, as well as the perceptions of its impact on performance, emerged as the main variable associated with the use of denial as coping strategy to deal with stress of the competition. More specifically, denial was more likely to be used when athletes felt higher anxiety, anger, and happiness. On the other hand, when dejection, excitement, and anger (only marginally significant) were perceived as more detrimental to performance, denial was also more likely to be strategy more frequently selected.

In a nutshell, results show that the use of problem-solving strategies (i.e., active coping), strategies to deal with emotions (i.e., emotional support and humor), and denial were explained by different mechanisms. Gender was related with the use of emotional support (for women) and humor (for men), while individual sports were associated with the use of emotional support. Overall stress did not significantly explain any of the coping strategies. Regarding cognitive appraisal, only higher perceptions of control and lower perceptions of coping emerged as related variables (for active coping and humor, respectively). Interestingly, primary cognitive appraisal did not explain the use of any adaptive or maladaptive coping strategies. Intensity of emotions emerged as the most significant set of related dimensions for the use of coping strategies, especially for denial. On the other hand, perceiving particular emotions as unhelpful for performance was associated with a higher use of emotional support, humor, and denial, but not active coping. Finally, the interaction between intensity and direction of emotions did not explain any of the coping strategies.

**Table 3:** Results from Hierarchical Regression (block 6).

Independent Variables	Active Coping			Emotional Support			Humor			Denial		
	*β*	*t*	*p*	*β*	*t*	*p*	*β*	*t*	*p*	*β*	*t*	*p*
Sex [0 = male]	0.03	0.65	0.519	0.11	1.98	**0.048**	−0.11	−1.99	**0.048**	−0.01	−1.91	0.058
Age	0.09	1.66	0.098	0.04	0.65	0.519	−0.05	−0.77	0.443	−0.05	−0.82	0.412
Type of sport [0 = individual]	−0.04	−0.61	0.542	−0.21	−3.00	**0.003**	−0.06	−0.89	0.373	0.07	1.04	0.300
**Coping-R Stress**												
Overall stress	0.07	1.24	0.216	0.09	1.61	0.109	−0.09	−1.59	0.112	−0.06	−1.16	0.248
**PSCAS: Cognitive appraisal**												
Threat	0.04	0.61	0.546	0.07	1.06	0.291	−0.03	−0.38	0.707	−0.04	−0.56	0.579
Challenge	0.11	1.74	0.082	0.04	0.56	0.579	−0.02	−0.27	0.785	−0.05	−0.80	0.426
Coping	−0.02	−0.27	0.789	−0.03	−0.51	0.613	−0.21	−3.12	**0.002**	−0.06	−0.99	0.322
Control	0.16	2.49	**0.013**	0.06	0.81	0.417	0.13	1.91	0.057	0.05	−0.81	0.418
**SEQ: Emotion intensity**												
Anxiety	−0.07	−0.96	0.337	−0.09	−1.19	0.236	−0.13	−10.74	0.084	0.18	2.56	**0.011**
Dejection	0.12	10.48	0.141	0.09	1.13	0.259	−0.02	−0.20	0.844	0.05	0.68	0.494
Anger	−0.04	−0.53	0.594	0.01	0.17	0.865	0.24	3.18	**0.002**	0.33	4.70	**<0.001**
Excitement	0.28	3.43	**0.001**	0.28	3.27	**0.001**	0.06	0.67	0.504	0.02	0.26	0.798
Happiness	−0.05	−0.60	0.549	0.11	1.21	0.228	0.11	1.17	0.243	0.23	2.68	**0.008**
**SEQ: Emotion direction**												
Anxiety	0.06	0.99	0.322	−0.01	−0.10	0.922	0.08	1.33	0.186	0.06	1.06	0.291
Dejection	0.08	0.82	0.411	0.02	0.18	0.855	0.13	1.31	0.191	−0.19	−2.06	**0.040**
Anger	−0.17	−1.73	0.084	0.01	0.03	0.975	−0.19	−1.88	0.061	0.18	1.91	0.057
Excitement	0.02	0.20	0.845	−0.16	−2.07	**0.039**	−0.02	−0.25	0.805	−0.17	−2.35	**0.019**
Happiness	0.10	1.30	0.195	0.08	1.08	0.279	−0.15	−2.02	**0.044**	−0.10	−1.36	0.175
**SEQ: Emotion Intensity** × **Direction interaction**												
Anxiety	−0.07	−1.42	0.158	0.09	1.62	0.107	−0.06	−1.09	0.276	0.01	0.24	0.807
Dejection	−0.09	−1.59	0.112	−0.02	−2.89	0.773	0.01	0.22	0.829	0.06	1.00	0.319
Anger	0.05	0.87	0.384	0.06	1.04	0.300	0.03	0.50	0.620	−0.05	−0.97	0.322
Excitement	−0.05	−0.98	0.329	−0.10	−1.89	0.060	0.02	0.42	0.673	−0.02	−0.36	0.721
Happiness	0.08	1.48	0.140	0.10	1.77	0.078	−0.03	−0.55	0.584	−0.03	−0.58	0.564
***F*(*df*), *p***	4.06 (23,354) < 0.001			3.23 (23,354) < 0.001			2.98 (23,350) < 0.001			5.32 (23,354) < 0.001		
***R*^2^(*R*^2^*Aj*.)**	0.21 (0.16)			0.17 (0.12)			0.16 (0.11)			0.26 (0.21)		

## 4. Discussion

This study analyzed the intention to use coping strategies as central elements of sporting adaptation to a stressful situation defined as “not achieving the desired performance in the next competition”, in high-level athletes who compete at the top national leagues in their sports. The results can be summarized into six main aspects.

First, regarding problem-solving strategies, which the literature has confirmed as the dimension most reported by athletes [6], the model confirmed the explanatory importance of positive emotions (e.g., excitement), as suggested in other studies [18,19] and greater control of the situation [37]. Thus, increased power over their own actions leads athletes to act on the problem, a fact that confirms the need for psychological practice that focuses on increasing the perception of control [30]. Resultingly, such a profile may facilitate performance [42].

Second, the main independent variables of the use of emotional support were gender, the type of sport, excitement, and the feeling that excitement has a facilitative effect on performance. Thus, it was observed that, in line with the literature, female athletes reported a higher intention to use these strategies [43]. It should also be noted that the more regular use of emotional support from others or coaches was explained by the practice of individual sports, a fact that could be studied more extensively in future studies. Also noteworthy is the close relationship that emerges between emotional support and excitement, and it should be emphasized that coping dimensions based on emotional management may be associated with typically more positive emotional patterns that promote higher performance [11,13]. Overall, the emotional support approach may be considered as an adaptive strategy, but helping athletes to reconsider excitement as facilitative for performance may lead to a greater adoption of active coping strategies which directly confront a stressor rather than focusing only on the emotional response.

Third, humor was reported more by male athletes. Humor was associated with lower coping perceptions and higher anxiety intensity, suggesting that it is a coping strategy that allows males to deal with stress. Although this may be helpful in some respects such as offering a way to turn down the dial of anger, questions remain over whether humor is a strategy that is always conducive to actively addressing problems related to stressful situations.

Fourth, the use of denial was more frequent among male athletes, which can be explained by the fact that male athletes reported more typically unhelpful emotions (e.g., anxiety and anger) but also reported higher levels of joy and attributed a more positive effect to anger and a less positive effect to dejection and excitement. As a dimension focused on passive emotion regulation, which is conceptually close to dimensions such as withdrawal in the approach of Nicholls et al. [5], the regular use of this strategy may allow athletes to protect themselves from the negative impact of emotions. However, according to research data, the use of denial is not an indicator of successful adjustment to competition [5]. The finding that when athletes experience dejection, they will likely use denial as a coping strategy may typify an avoidance approach toward motivation. Stress theories suggest that such an approach is an antecedent of threat appraisals, which means that performing well is less likely to occur [29]. Guiding athletes from a coping strategy of initial denial to more active coping strategies could be fruitful in terms of performance and wellbeing. Indeed, strategies rooted in Rational Emotive Behavior Therapy (REBT; [44]) may facilitate rational and adaptive beliefs about adversity. For example, REBT can reduce unhealthy negative emotional states such as depression and promote healthy negative emotions such as sadness when facing adversity [45].

Fifth, this study confirms the importance of personal and sporting variables in explaining athletes’ ability to cope. Regarding gender differences, it was found that male athletes resort more frequently to denial and humor, and attribute greater effectiveness to the use of coping. On the other hand, female athletes reported more regular use of emotional support. Thus, the literature has highlighted that male athletes resort more regularly to problem-solving coping and are more effective in their use and, on the other hand, female athletes opt more habitually for emotion-focused strategies [46]. Athletes’ age did not prove to be decisive in the final configuration of the models tested; however, correlations showed that older and/or more experienced athletes reported stronger intention to use problem-solving more coping strategies. This result can perhaps be explained by drawing on their previous success in coping with stressful situations or by being guided by a generally more adaptive view of stress [30,47]. Regarding to the type of sport, this variable proved to be important in explaining the greater use of emotional support and humor observed in individual athletes.

Sixth, a final aspect relates to the level of stress attributed to the possibility of not performing as expected in the next competition. Although the level of stress was not a significant variable in the final formulation of the models tested, it is worth noting that the increase in stress, as indicated by correlations (and regression analyses when considering only the first two blocks of the applied regression model) was associated with a greater intention the use of active confrontation and emotional support, whereas in the opposite direction, the attribution of less stress about the performance situation described in this study was related to a greater intention to use humor.

In sum, coping can be successful in mitigating or eliminating the negative impact of the evaluation of the stressful situation, and it has become clear that cognitive evaluation processes are decisive in understanding the intention to use coping and the effectiveness of its use. Thus, while the perception of control emerged as an important variable in explaining a greater intention to use problem-focused strategies, the perception of coping is fundamental in explaining the achievement of greater coping effectiveness since both models are related to the promotion of superior sports performance [1]. Indeed, both control and coping perceptions helped to explain the more regular choice of active emotional regulation. Another aspect to highlight is the fact that emotional management has emerged as an essential process for understanding the intention to use the different coping dimensions, as well as the effectiveness attributed to this intention [48]. More specifically, it was found that attributing a more facilitative effect to negative emotions such as anxiety and anger is associated with the use of problem-solving and active emotional regulation strategies, confirming the importance of this more active confrontational stance in the way emotions are interpreted [13,20,24].

In conclusion, we share the assertion that cognitive appraisal, emotions, and coping are highly related constructs, and that both cognitive appraisal and emotions are important as determinants of the intention to use coping [9,15,18,19,20]. Ultimately, there is never only one way to cope with a stressful situation, and building a repertoire of coping strategies may be more effective and versatile than relying only on one.

## 5. Main Conclusions

The results partially supported our hypothesis. Coping strategies were associated with psychological, sport-related, and personal factors, with problem-focused coping and higher coping efficacy linked to positive psychological variables (e.g., positive emotions, facilitative emotional direction, and functional appraisals such as challenge and control). However, coping strategies such as emotional support, humor, and denial, showed different patterns, suggesting that the relationship between these constructs is more complex than anticipated and may rely on different mechanisms.

### 5.1. Limitations and Future Directions

The present study has several methodological strengths: it included a relatively large sample of high-level athletes, derived from different clubs; quality of data was ensured by face-to-face data collection; a critical incident approach allowed researchers to ensure that the upcoming competition was salient in athletes’ minds; and, finally, instruments validated to the Portuguese population were used. Regardless of its strengths, some limitations need to be acknowledged and discussed. First, the Reduced Coping Inventory (Coping-R [39]) defined a specific situation as stressor. We recognize that different athletes will be stressed by different situations, but this confounding effect was eliminated by using the same stressor for all of them. However, this also eliminated the richness of individual experiences. Moreover, the dimension of denial presented a lower internal consistency than expected that can be explained by the fact that athletes were confronted with an hypothetical situation that hopefully they expect to not occur. Even though this measure has been used in prior studies, its factorial structure should be reexamined.

Another important limitation concerns the absence of detailed information on training volume and recent performance outcomes. Although all athletes were high-level competitors participating in national leagues in their respective sports, we did not systematically collect quantitative data regarding weekly training load or objective performance metrics. These variables could provide a richer contextual understanding of coping processes and serve as valuable covariates in future research. Therefore, upcoming studies should incorporate such information to enable more comprehensive modeling and better control of potential influencing variables.

In future studies, the relationship between the constructs that make up the process of adaptation to stress and the results of this adjustment should be studied in greater depth, namely sporting performance and satisfaction with the levels of performance achieved by athletes [11,46]. Equally important will be the adoption of longitudinal designs and the collection of idiosyncratic data (e.g., recording through the think-aloud technique [6,49], to broaden our knowledge of how athletes deal with the same stressful situation over time, understanding whether situational or dispositional approaches to coping make more sense [5,20,50].

### 5.2. Implications for Practice

In terms of implications for practice, it is important to expand our knowledge of the stressors that occur most frequently in sports contexts and that are most likely to cause high levels of disturbance in athletes, so that training programs can be supported to reduce the harmful nature of these stressors [1,51]. To illustrate, self-compassion is one such technique that may promote more adaptive emotions and coping strategies [52]. We should also remember that sport is a highly social activity involving continual influence of coaches, team-mates and support staff [53]. Therefore, enhancing education about the interplay of emotions, coping, and cognitive appraisal on a social and cultural level is of importance. Awareness of when particular coping strategies may be most effective should also be promoted when working with athletes through reflection. For example, when the stressor is controllable, problem-focused coping may be effective, but when little can be done to alter a situation, emotion-focused coping may be more beneficial [7]. Also important, our study can have implications for adults surrounding athletes activity. For example, coaches should be aware that the possibility of “not achieving the desired performance in the upcoming competition” is stressful for athletes and that this situation is perceived and coped differentially by athletes according their gender, age, ant type of sport; probably one major implication for coaches is that they should discuss and prepare athletes for this negative situation analyzing how they can proceed when facing undesirable performance during and after competitions (eventually, coaches are much more motivated to prepare their athletes to be successful in competitions, disregarding the opposite scenario). For sport psychologists, it is also important to design psychological interventions directed to promote cognitions, emotions, and behaviors related to how to face the possibility of not performing well during and after competitions, existing several strategies that can help athletes to be well prepared for competitions [54]. Even for significant others (families, friends, teammates, among others), there are also implications due the need to help athletes to cope with unsuccessful performance, providing emotional support and help them to “ventilate” their feelings and thoughts, preventing the exposure to psychological problems (see, for example, [55]).

## Figures and Tables

**Table 1:** Skewness, kurtosis, VIF, Means (SD) and correlations between variables of the study.

	|sk|	|ku|	VIF	1.	2.	3.	4.	5.	6.	7.	8.	9.	10.	11.	12.	13.	14.	15.	16.	17.	18.
**Coping-R Coping**																					
1. Active coping	0.51	0.28	1.50	3.8 (0.8)																	
2. Emotional s	0.03	0.55	1.37	0.42 ***	3.0 (0.9)																
3. Humor	1.16	0.93	1.26	−0.09	0.07	1.9 (0.9)															
4. Denial	1.14	0.47	1.44	−0.16 **	0.11 *	0.31 ***	1.6 (0.7)														
**Coping-R Stress**																					
5. Overall stress	0.32	0.47	1.31	0.11 *	0.09	−0.19 ***	0.02	3.4 (1.1)													
**PSCAS: C. appr.**																					
6. Threat	0.04	0.51	1.83	0.09	0.10	−0.11 *	0.12 *	0.41 ***	4.5 (1.4)												
7. Challenge	1.19	1.50	1.41	0.20 ***	0.21 ***	−0.11 *	−0.01	0.23 ***	0.35 ***	6.1 (1.0)											
8. Coping	0.76	0.69	1.75	0.22 ***	0.07	−0.09	−0.10	−0.06	−0.13 *	0.18 ***	5.6 (0.9)										
9. Control	0.32	0.47	1.41	0.21 ***	0.18 ***	0.12 *	−0.09	−0.13 *	−0.12 *	0.07	0.37 ***	4.9 (1.3)									
**SEQ: Emotions-I**																					
10. Anxiety(I)	0.14	0.56	2.22	0.08	0.13 *	−0.10 *	0.17 ***	0.41 ***	0.60 ***	0.32 ***	−0.24 ***	−0.06	1.7 (0.9)								
11. Dejection(I)	2.60	7.49	2.56	−0.06	−0.05	0.08	0.26 ***	0.07	0.15 **	−0.11 *	−0.21 ***	−0.27 ***	0.14 **	0.4 (0.7)							
12. Anger(I)	2.51	6.17	2.29	0.01	0.01	0.11 *	0.34 ***	0.09	0.24 ***	0.06	−0.07	−0.14 **	0.23 ***	0.69 ***	0.4 (0.7)						
13. Excitement(I)	0.31	0.32	2.95	0.34 ***	0.27 ***	−0.02	0.03	0.10 *	0.09	0.34 ***	0.35 ***	0.17 ***	0.28 ***	−0.29 ***	−0.06	2.3 (0.8)					
14. Happiness(I)	0.66	0.02	3.44	0.19 ***	0.23 ***	−0.01	−0.01	−0.02	−0.06	0.27 ***	0.22 ***	0.23 ***	0.08	−0.51 ***	−0.28 ***	0.59 ***	2.6 (1.0)				
**SEQ: Emotions-D**																					
15. Anxiety(D)	0.18	0.41	1.44	0.12 *	0.04	0.06	−0.01	−0.07	−0.04	0.04	0.29 ***	0.19 ***	−0.09	−0.11 *	−0.03	0.21 ***	0.08	0.2 (1.0)			
16. Dejection(D)	0.38	0.43	3.78	0.06	0.03	0.02	0.14 **	−0.03	−0.02	0.03	0.13 **	0.09	−0.09	−0.17 ***	−0.14 **	0.15 **	0.16 **	0.46 ***	0.1 (1.3)		
17. Anger(D)	0.25	0.97	3.81	0.06	0.06	0.01	−0.09	−0.04	0.03	0.11 *	0.15 **	0.14 **	−0.04	−0.12 *	−0.01	0.17 ***	0.13 *	0.47 ***	0.84 ***	0.2 (2.0)	
18. Excitement(D)	0.60	0.42	2.24	0.29 ***	0.13 *	−0.10	−0.17 ***	0.07	−0.01	0.18 ***	0.32 ***	0.14 **	0.08	−0.21 ***	−0.08	0.57 ***	0.31 ***	0.23 ***	0.18 ***	0.20 ***	1.3 (0.9)
19. Happiness(D)	0.46	0.06	2.62	0.20 ***	0.15 **	−0.11 *	−0.11 *	0.06	−0.03	0.19 ***	0.20 ***	0.13 **	0.03	−0.33 ***	−0.18 ***	0.37 ***	0.66 ***	0.07	0.18 ***	0.18 ***	1.5 (1.0)

Note. I = intensity; D = direction; * *p* < 0.05; ** *p* < 0.01; *** *p* < 0.001.

**Table 2:** Summary of main results from Hierarchical Regression Analyses.

Independent Variables	Active Coping	Emotional Support	Humor	Denial
Sex	No	**Yes**	**Yes**	No
Age	No	No	No	No
Type of sport	No	**Yes**	No	No
Overall stress	No	No	No	No
**Cognitive appraisal**				
Threat	No	No	No	No
Challenge	No	No	No	No
Coping	No	No	**Yes (−)**	No
Control	**Yes (+)**	No	No	No
**Intensity**				
Anxiety	No	No	No	**Yes (+)**
Dejection	No	No	No	No
Anger	No	No	**Yes (+)**	**Yes (+)**
Excitement	**Yes (+)**	**Yes (+)**	No	No
Happiness	No	No	No	**Yes (+)**
**Direction**				
Anxiety	No	No	No	No
Dejection	No	No	No	**Yes (−)**
Anger	No	No	**Yes (+)**	**Yes (−)**
Excitement	No	**Yes (+)**	No	**Yes (−)**
Happiness	No	No	**Yes (+)**	No

Note: Each row indicates whether the independent variable emerged as significant or not. In the cases of significant associations, the signs “(+)” and “(−)” represent a positive or negative association, respectively.

## Data Availability

The raw data supporting the conclusions of this article will be made available by the authors on request.

## Supplementary Materials

The following supporting information can be downloaded at: https://www.mdpi.com/article/10.3390/sports13100366/s1, Table S1: Descriptive statistics based on Sex and Type of Sport; Table S2a: Results from Hierarchical Regression. Dependent Variable: Active Coping; Table S2b: Results from Hierarchical Regression. Dependent Variable: Emotional Support; Table S2c: Results from Hierarchical Regression. Dependent Variable: Humor; Table S2d: Results from Hierarchical Regression. Dependent Variable: Denial.
