# Supplementary material for: Coping Strategies Before Competition: The Role of Stress, Cognitive Appraisal, and Emotions
Source: Sports (Basel). 2025 Oct 16;13(10):366. doi: 10.3390/sports13100366 (PMC12568298; doi:10.3390/sports13100366)
Supplement: Supplementary file 1 [file sports-13-00366-s001.zip › sports-3796230-supplementary.pdf]

sports

Table S1. ***Descriptive statistics based on Sex and Type of Sport.***

	**Sex**		**Type of sport**		**Total sample**
	Male<br>M(SD)	Female<br>M(SD)	Individual<br>M(SD)	Team<br>M(SD)	M(SD)
Overall stress	3.2(1.1)	3.6(1.1)	3.3(1.1)	3.4(1.1)	3.4(1.1)
**Coping strategies**					
Active coping	3.8(0.8)	3.8(0.8)	3.8(0.9)	3.8(0.7)	3.8(0.8)
Emotional support	2.9(0.9)	3.1(0.9)	3.2(0.9)	2.9(0.9)	3.0(0.9)
Humor	2.0(1.0)	1.8(0.9)	2.1(1.0)	1.9(0.9)	1.9(0.9)
Denial	1.8(0.7)	1.7(0.6)	1.8(0.7)	1.8(0.7)	1.6(0.7)
**Cognitive appraisal**					
Threat	4.4(1.2)	4.6(1.2)	4.3(1.1)	4.6(1.2)	4.5(1.2)
Challenge	6.1(0.9)	6.2(0.8)	6.1(0.8)	6.2(0.8)	6.1(0.8)
Coping	5.8(0.9)	5.4(1.0)	5.4(1.0)	5.8(0.9)	5.6(0.9)
Control	4.9(1.3)	4.8(1.3)	5.6(1.0)	4.3(1.3)	4.9(1.3)
**Emotions' intensity**					
Anxiety	1.6(0.9)	1.8(0.9)	1.8(0.9)	1.6(0.9)	1.7(0.9)
Dejection	0.4(0.7)	0.3(0.6)	0.2(0.5)	0.4(0.7)	0.4(0.7)
Anger	0.4(0.8)	0.3(0.6)	0.2(0.6)	0.5(0.8)	0.4(0.7)
Excitement	2.3(0.8)	2.1(0.8)	2.2(0.8)	2.4(0.8)	2.3(0.8)
Happiness	2.5(1.1)	2.7(0.9)	2.6(0.9)	2.5(1.1)	2.6(1.0)
**Emotions' direction**					
Anxiety	0.3(0.9)	-0.1(1.0)	0.1(1.0)	0.2(1.0)	0.2(1.0)
Dejection	0.1(1.2)	0.1(1.4)	0.0(1.2)	0.2(1.4)	0.1(1.3)
Anger	0.2(1.1)	0.2(1.3)	0.2(1.1)	0.2(1.2)	0.2(1.2)
Excitement	1.3(0.9)	1.3(0.9)	1.2(0.9)	1.4(0.9)	1.3(0.9)
Happiness	1.4(1.0)	1.7(0.9)	1.4(1.0)	1.6(1.0)	1.5(1.0)

sports MDPI

Table S2a. ***Results from Hierarchical Regression. Dependent Variable: Active Coping.***

Variables	*Block 1*			*Block 2*			*Block 3*			*Block 4*			*Block 5*			*Block 6*		
	β	*t*	*p*	*β*	*t*	*p*	*β*	*t*	*p*	*β*	*t*	*p*	*β*	*t*	*p*	*β*	*t*	*p*
Sex [0 = male]	.017	.324	.746	-.004	-.067	.946	.013	.249	.804	.041	.810	.418	.032	.626	.532	.033	.646	.519
Age	.159	2.76	**.006**	.159	2.79	**.006**	.080	1.40	.163	.084	1.54	.125	.084	1.50	.135	.094	1.66	.098
Type of sport [individual]	-.059	-1.04	.300	-.068	-1.20	.231	.033	.492	.623	-.021	-.307	.759	-.035	-.514	.608	-.041	-.610	.542
Coping-R: Overall stress				.113	2.17	**.030**	.094	1.73	.084	.073	-1.35	.178	.063	1.51	.250	.068	1.24	.216
PCAS: Threat perception							.025	.424	.672	.056	.834	.405	.068	1.01	.314	.042	.605	.546
PCAS: Challenge perception							.152	2.69	**.008**	.104	1.69	.092	.105	1.69	.092	.108	1.74	.082
PCAS: Coping perception							.086	1.40	.162	.014	.223	.824	.005	.076	.939	-.018	-.267	.789
PCAS: Control perception							.184	2.77	**.006**	.162	2.49	**.013**	.164	2.49	**.013**	.164	2.49	**.013**
SEQ: Anxiety (Intensity)										-.078	-1.12	.265	-.081	-1.15	.249	-.068	-.962	.337
SEQ: Dejection (Intensity)										.134	1.79	.078	.123	1.61	.107	.115	1.48	.141
SEQ: Anger (Intensity)										.049	-.710	.478	-.032	-.448	.654	-.038	-.533	.594
SEQ: Excitement (Intensity)										.311	4.72	**<.001**	.310	3.77	**<.001**	.284	3.43	**.001**
SEQ: Happiness (Intensity)										-.017	-.248	.804	-.082	-.947	.344	-.053	-.600	.549
SEQ: Anxiety (Direction)													.027	.472	.637	.058	.993	.322
SEQ: Dejection (Direction)													.062	.672	.502	.078	.824	.411
SEQ: Anger (Direction)													-.116	-1.26	.209	-.165	-1.73	.084
SEQ: Excitement (Direction)													.015	.206	.837	.015	.195	.845
SEQ: Happiness (Direction)													.097	1.32	.188	.096	1.30	.195
SEQ: Anxiety (Interaction)																-.073	-1.42	.158
SEQ: Dejection (Interaction)																-.093	-1.59	.112
SEQ: Anger (Interaction)																.050	.871	.384
SEQ: Excitement (Interaction)																-.053	-.978	.329
SEQ: Happiness (Interaction)																.081	1.48	.140
F(gl), p	2.55 (3, 374), .055			3.11 (4, 373), .015*			6.17 (8, 369), <.001***			6.16 (13,364), <.001***			4.69 (18,359), <.001***			4.06 (23,354), <.001***		
ΔF, p	2.55, .055			4.72, .030*			8.97, <.001***			5.54, <.001***			.890, .488			1.64, .149		
ΔR²	.020			.012			.086			.062			.010			.018		
R²(R²Aj.)	.020 (.012)			.032 (.022)			.118 (.099)			.180 (.151)			.190 (.150)			.209 (.157)		

*Nota: *p< .05. **p<.01. ***p< .001.*

Table S2b. *Results from Hierarchical Regression. Dependent Variable: Emotional Support.*

Variables	*Block 1*			*Block 2*			*Block 3*			*Block 4*			*Block 5*			*Block 6*		
	β	***t***	***p***	***β***	***t***	***p***	***β***	***t***	***p***	***β***	***t***	***p***	***β***	***t***	***p***	***β***	***t***	***p***
Sex	.088	1.71	.089	.067	1.28	.202	.072	1.37	.171	.083	1.59	.114	.090	1.69	.091	.105	1.98	**.048**
Age	.065	1.14	.256	.065	1.16	.248	.026	.446	.656	.044	.774	.440	.037	.649	.517	.037	.646	.519
Type of sport	-.188	-3.35	**.001**	-.198	-3.53	**<.001**	-.162	-2.37	**.018**	-.215	-3.13	**.002**	-.209	-3.02	**.003**	-.207	-3.00	**.003**
Coping-R: Overall stress				.118	2.30	**.022**	.085	1.53	.126	.077	1.38	.168	.078	1.39	.164	.090	1.61	.109
PCAS: Threat perception							.045	.750	.454	.086	1.26	.207	.080	1.15	.252	.074	1.06	.291
PCAS: Challenge perception							.135	2.35	**.020**	.040	.640	.523	.041	.648	.517	.035	.555	.579
PCAS: Coping perception							.043	.689	.491	-.009	-.136	.892	-.001	-.019	.985	-.034	-.507	.613
PCAS: Control perception							.071	1.06	.292	.042	.628	.530	.044	.659	.510	.055	.812	.417
SEQ: Anxiety (Intensity)										-.063	-.888	.375	-.072	-1.00	.316	-.086	-1.19	.236
SEQ: Dejection (Intensity)										.105	1.36	.175	.095	1.23	.220	.090	1.13	.259
SEQ: Anger (Intensity)										.027	.384	.702	.021	.294	.769	.012	.170	.865
SEQ: Excitement (Intensity)										.194	2.88	**.004**	.287	3.41	**.001**	.277	3.27	**.001**
SEQ: Happiness (Intensity)										.150	2.18	**.030**	.087	.985	.325	.108	1.21	.228
SEQ: Anxiety (Direction)													.008	.144	.885	-.006	-.098	.922
SEQ: Dejection (Direction)													-.044	-.473	.637	.018	.183	.855
SEQ: Anger (Direction)													.030	.323	.747	.003	.032	.975
SEQ: Excitement (Direction)													-.137	-1.84	.066	-.158	-2.07	**.039**
SEQ: Happiness (Direction)													.070	.929	.354	.082	1.08	.279
SEQ: Anxiety (Interaction)																.085	1.62	.107
SEQ: Dejection (Interaction)																-.017	-2.89	.773
SEQ: Anger (Interaction)																.061	1.04	.300
SEQ: Excitement (Interaction)																-.104	-1.89	.060
SEQ: Happiness (Interaction)																.099	1.77	.078
F (gl), p	5.10 (3,374),.002			5.20 (4,373), <.001			4.55 (8,369), <.001			4.73 (13,364), <.001			3.61 (18,359), <.001			3.23 (23,354), <.001		
ΔF, p	5.11, .002			5. 30, .022			3.75, .005			4.65, <.001			.743, .591			1.74, .124		
ΔR²	.039			.013			.037			.055			.009			.020		
R²(R²Aj.)	.039 (.032)			.053 (.043)			.090 (.070)			.144 (.114)			.153 (.111)			.174 (.120)		

Table S2c. *Results from Hierarchical Regression. Dependent Variable: Humor.*

Variables	*Block 1*			*Block 2*			*Block 3*			*Block 4*			*Block 5*			*Block 6*		
	β	***t***	***p***	***β***	***t***	***p***	***β***	***t***	***p***	***β***	***t***	***p***	***β***	***t***	***p***	***β***	***t***	***p***
Sex	-.159	-3.05	**.002**	-.133	-2.53	**.012**	-.155	-2.96	**.003**	-.130	-2.45	**.015**	-.102	-1.92	.055	-.107	-1.99	**.048**
Age	-.058	-1.02	.310	-.058	-1.03	.303	-.033	-.568	.570	-.031	-.537	.592	-.041	-.707	.480	-.045	-.768	.443
Type of sport	-.122	-2.17	**.031**	-.110	-1.97	**.050**	-.015	-.223	.824	-.062	-.893	.372	-.060	-.870	.385	-.062	-.892	.373
Coping-R: Overall stress				-.142	-2.76	**.006**	-.105	-1.90	.058	-.091	-1.62	.107	-.088	-1.57	.118	-.090	-1.59	.112
PCAS: Threat perception							-.057	-.965	.335	-.044	-.640	.522	-.030	-.435	.664	-.027	-.376	.707
PCAS: Challenge perception							.034	-.589	.556	-.033	-.521	.603	-.021	-.338	.736	-.018	-.274	.785
PCAS: Coping perception							-.214	-3.44	**.001**	-.239	-3.68	**<.001**	-.234	-3.59	**<.001**	-.213	-3.12	**.002**
PCAS: Control perception							.145	2.14	**.033**	.137	2.03	**.043**	.140	2.08	**.038**	.131	1.91	.057
SEQ: Anxiety (Intensity)										-.114	-1.59	.113	-.132	-1.83	.068	-.127	-1.74	.084
SEQ: Dejection (Intensity)										-.028	-.356	.722	-.033	-.416	.677	-.016	-.197	.844
SEQ: Anger (Intensity)										.219	3.07	**.002**	.247	3.39	**.001**	.238	3.18	**.002**
SEQ: Excitement (Intensity)										.046	.679	.497	.062	.735	.463	.057	.669	.504
SEQ: Happiness (Intensity)										.019	.273	.785	.112	1.26	.208	.106	1.17	.243
SEQ: Anxiety (Direction)													.065	1.11	.267	.081	1.33	.186
SEQ: Dejection (Direction)													.140	1.49	.138	.129	1.31	.191
SEQ: Anger (Direction)													-.180	-1.90	.058	-.185	-1.88	.061
SEQ: Excitement (Direction)													-.028	-.375	.708	-.019	-.248	.805
SEQ: Happiness (Direction)													-.154	-2.04	**.042**	-.154	-2.02	.044
SEQ: Anxiety (Interaction)																-.058	-1.09	.276
SEQ: Dejection (Interaction)																.013	.217	.829
SEQ: Anger (Interaction)																.030	.497	.620
SEQ: Excitement (Interaction)																.024	.422	.673
SEQ: Happiness (Interaction)																-.031	-.548	.584
F***(gl), p***	5.20 (3,370), .002			5.88 (4,369), <.001			4.89 (8,365), <.001			4.26 (13,360), <.001			3.74 (18,355), <.001			2.98 (23,350), <.001		
ΔF, p	5.20, .002			7.63, .006			3.72, .006			3.04, .011			2.20, .054			.355, .879		
ΔR^2	.040			.019			.037			.037			.026			.004		
$R^2(R^2Aj.)$	.040 (.033)			.060 (.050)			.097 (.077)			.133 (.102)			.159 (.117)			.164 (.109)		

Table S2d. *Results from Hierarchical Regression. Dependent Variable: Denial.*

**Variables**	***Block 1***			***Block 2***			***Block 3***			***Block 4***			***Block 5***			***Block 6***		
	β	***t***	***p***	***β***	***t***	***p***	***β***	***t***	***p***	***β***	***t***	***p***	***β***	***t***	***p***	***β***	***t***	***p***
Sex	-.133	-2.54	**.011**	-.132	-2.48	**.014**	-.155	-2.90	**.014**	-.123	-2.46	**.014**	-.095	-1.90	.058	-.096	-1.91	.058
Age	-.106	-1.85	.066	-.106	-1.84	.066	-.080	-1.34	.180	-.039	-.719	.472	-.046	-.858	.392	-.0.45	-.821	.412
Type of sport	.040	.701	.484	.040	.709	.479	.066	.943	.346	.043	.652	.515	.068	1.04	.301	.068	1.04	.300
Coping-R: Overall stress				-.007	-.144	.886	-.050	-.887	.375	-.083	-1.56	.121	-.065	-1.23	.219	-.061	-1.16	.248
PCAS: Threat perception							.117	1.93	.055	.036	-.549	.584	-.053	-.814	.416	-.037	-.555	.579
PCAS: Challenge perception							-.012	-.199	.843	-.043	-.703	.483	-.042	-.698	.486	-.048	-.798	.426
PCAS: Coping perception							-.139	-2.19	**.029**	-.081	-1.31	.190	-.072	-1.16	.245	-.064	-.991	.322
PCAS: Control perception							.051	.734	.463	.052	.809	.419	.047	.746	.456	.052	-.811	.418
SEQ: Anxiety (Intensity)										.201	2.94	**.003**	.189	2.79	**.006**	.176	2.56	**.011**
SEQ: Dejection (Intensity)										.049	.660	.510	.057	.784	.434	.052	.684	.494
SEQ: Anger (Intensity)										.348	5.19	**<.001**	.318	4.71	**<.001**	.327	4.70	**<.001**
SEQ: Excitement (Intensity)										-.083	-1.29	.197	.006	.072	.943	.021	.256	.798
SEQ: Happiness (Intensity)										.180	2.74	**.006**	.241	2.90	**.004**	.228	2.68	**.008**
SEQ: Anxiety (Direction)													.066	1.22	.225	.060	1.06	.291
SEQ: Dejection (Direction)													-.167	-1.89	.060	-.190	-2.06	.040
SEQ: Anger (Direction)													.141	1.59	.113	.176	1.91	.057
SEQ: Excitement (Direction)													-.156	-2.22	**.027**	-.170	-2.35	**.019**
SEQ: Happiness (Direction)													-.099	-1.40	.163	-.097	-1.36	.175
SEQ: Anxiety (Interaction)																.012	.244	.807
SEQ: Dejection (Interaction)																.057	.997	.319
SEQ: Anger (Interaction)																-.054	-.971	.322
SEQ: Excitement (Interaction)																-.019	-.357	.721
SEQ: Happiness (Interaction)																-.031	-.577	.564
F***(gl), p***	2.89 (3,374), .036			2.17 (4,373), .072			2.43 (8,369), .014			7.63 (13,364). <.001			6.71 (18,359), <.001			5.32 (23,354), <.001		
ΔF, p	2.89*, .036			.021, .886			2.65, .033			15.21, <.001			3.59, .003			.497, .779		
ΔR^2	.023			.000			.027			.104			.037			.005		
$R^2(R^2Aj.)$	.023 (.015)			.023 (.012)			.050 (.029)			.214 (.186)			.252 (.214)			.257 (.209)		
